# A Two‐Step Strategy Utilising Fluoroscopy for Resolving Peripheral Airway Impaction by an Aspirated Capsule Endoscope: A Case Report

**DOI:** 10.1002/rcr2.70600

**Published:** 2026-04-24

**Authors:** Misa Oguma, Ryo Nonomura, Kazunori Ueda, Hideyuki Nagata, Ryuga Yabe, Yutaka Oshima, Takanobu Sasaki, Naoya Ishibashi, Hiromichi Niikawa

**Affiliations:** ^1^ Postgraduate Training Center Tohoku Medical and Pharmaceutical University Hospital Sendai Japan; ^2^ Department of Thoracic Surgery Tohoku Medical and Pharmaceutical University Hospital Sendai Japan

**Keywords:** bronchoscopy, capsule endoscopy, case report, fluoroscopy, foreign body aspiration

## Abstract

Capsule endoscopy (CE) aspiration is extremely rare (0.003%–0.013%) but potentially life‐threatening. The CE is structurally challenging for standard bronchoscopic removal due to its large size, smooth surface and nonmetallic composition. While most aspirated capsules remain in the central airways, reports on peripheral impaction are limited. We report a case in which a CE was impacted deep within the right basal segmental bronchus of a man in his 70s. Conventional forceps manipulation failed because the capsule was completely impacted, preventing visibility beyond the obstruction. We successfully employed a two‐step strategy: (1) disimpaction using a balloon catheter guided by fluoroscopy and (2) retrieval in the central airway using a retrieval loop net. This fluoroscopy‐assisted two‐step strategy provides a safe and reliable approach for retrieving structurally challenging foreign bodies in the peripheral airways.

## Graphical abstract

In a rare case in which the capsule was endoscopically impacted deep in the peripheral airway, it was successfully removed using a two‐step strategy. This combined fluoroscopy‐guided manipulation in the peripheral airway with capture using a retrieval loop net in the central airway. This technique is valuable for extracting smooth foreign bodies that are difficult to grasp.

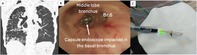

## 1 Introduction

Capsule endoscopy (CE) is a minimally invasive examination widely used primarily for the evaluation of small bowel diseases, and its safety is well established [1]. It is generally considered to have a low incidence of adverse events, with aspiration of a capsule endoscope being extremely rare (0.003%–0.013%) [1, 2]. Although aspiration itself is rare, the actual number of cases may increase because of the expanding patient base. It has also been reported that older men with comorbidities have a relatively higher risk of aspiration [1, 3]. Once aspiration occurs, it can be a life‐threatening complication requiring immediate intervention [3, 4].

The general approach for extracting an aspirated airway foreign body is bronchoscopy. However, a capsule endoscope is large and has a smooth surface, making it difficult to grasp. Furthermore, because it is nonmetallic, magnetic devices cannot be used, rendering it unsuitable for standard foreign‐body removal procedures. A review by Thorndal C et al. [1] suggests that most aspirated capsules remain in the central airways, such as the right main bronchus, and can be removed using bag‐like devices such as the Roth Net [4]. However, cases in which the capsule migrates to and becomes impacted in the peripheral airways are extremely rare, and reports of treatment are limited.

In this case, the capsule endoscope migrated to the peripheral airway and became impacted, making removal impossible with conventional forceps manipulation. However, cases where the capsule migrates to and becomes impacted in the peripheral airways are extremely rare. Unlike central airway impactions, there is currently no established or standardised retrieval strategy for completely impacted capsules in the peripheral airways. Therefore, a two‐step strategy was adopted: disimpaction under fluoroscopic guidance and capsule retrieval in the central airway, which allowed for safe extraction. We report on the usefulness of this procedure and review the literature.

## 2 Case Report

The case was a male patient in his 70s with a history of total gastrectomy, cholecystectomy and chronic obstructive pulmonary disease (COPD). The smoking history was 49 pack‐years. Immediately after swallowing a small bowel capsule endoscope for anaemia workup, he developed pharyngeal discomfort and cough and his SpO_2_ was 94% on room air.

**FIGURE 1:**
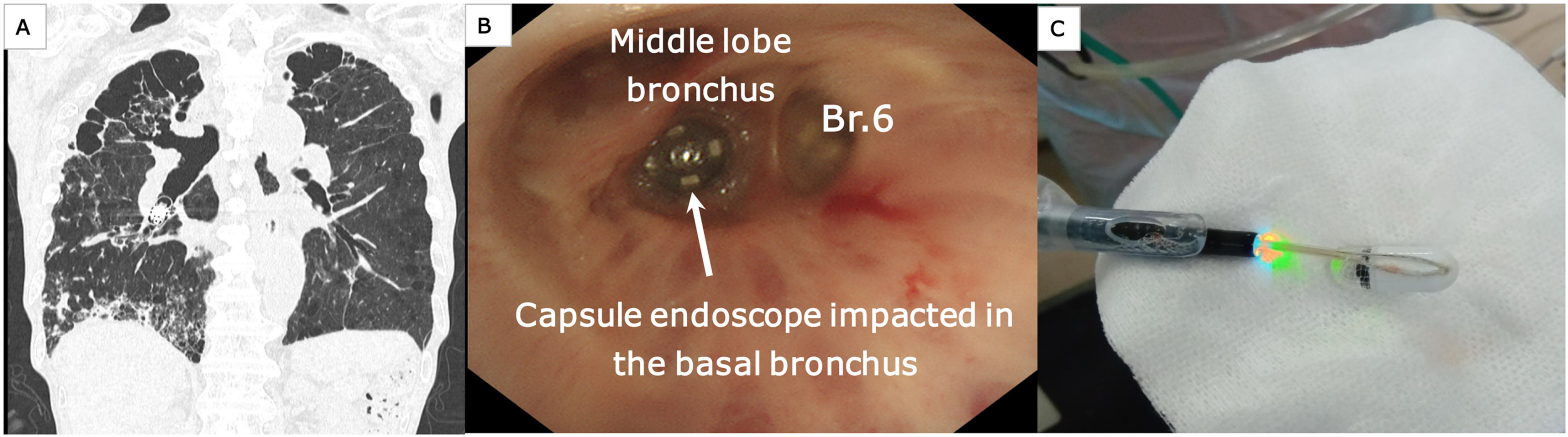
Impacted capsule endoscope. Plain chest CT (coronal view) reveals a foreign body in the right basal bronchus (A). A retained capsule endoscope is observed in the right basal bronchus (B). Retrieved/removed retained capsule endoscope (C).

A chest X‐ray revealed a foreign‐body shadow in the right mid‐lung field, prompting a referral to our department. A chest computed tomography (CT) scan confirmed a foreign body consistent with the capsule endoscope inside the right basal segmental bronchus, indicating that it was an airway foreign body (Figure 1A). Since coughing did not dislodge the object, removal was attempted under flexible bronchoscopy (1 T290).

The procedure was performed under conscious sedation using intravenous midazolam, with careful respiratory monitoring due to the patient's severe COPD. Upon observation, the vocal cords were incompletely closed. Advancing the scope, the capsule endoscope was observed in the right basal segment and was completely impacted, with no mobility even with induced coughing (Figure 1B). The distal side was not visible, and blind manipulation was deemed dangerous.

**FIGURE 2:**
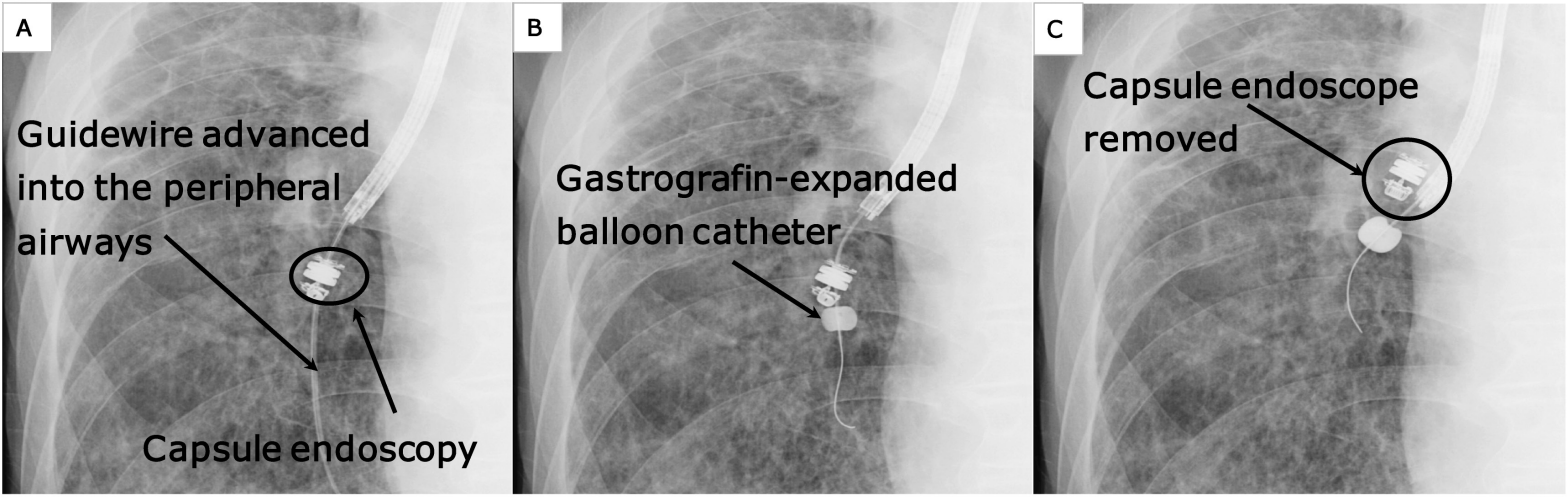
Procedure performed under fluoroscopic guidance. Guide the guidewire into the peripheral airways (A). The balloon was filled with gastrografin so that it could be seen under fluoroscopy (B). The retained capsule endoscope was retrieved together with the balloon catheter (C).

Therefore, a decision was made to safely carry out the removal procedure under fluoroscopic guidance. During the fluoroscopy‐guided balloon traction step, the flexible bronchoscope (1 T290) was positioned and stabilised at the proximal orifice of the truncus intermedius to ensure a stable field of view. First, a 0.025‐in. guidewire (Endoselector) was successfully passed through a minimal gap between the capsule and the bronchial wall (Figure 2A). A 4Fr double‐lumen disposable balloon catheter with a maximum inflation diameter of 11 mm (Model B5‐2C; Olympus, Tokyo, Japan) was subsequently advanced over the guidewire to the distal side of the capsule. The balloon was then inflated with approximately 1.5 mL of contrast medium (Gastrografin) under direct fluoroscopic visualisation until adequate resistance was felt against the bronchial wall (Figure 2B). While specific inflation pressure was not continuously monitored, careful manual inflation was performed to prevent airway mucosal injury. The inflated balloon was gently pulled proximally, dragging the capsule into the central airway (Figure 2C). Once the capsule reached the right main bronchus, it was securely captured and retrieved using an oval loop net (net size: 30 × 60 mm; catheter diameter: 2.5 mm; MED‐133‐NET; Meditalia, Italy) (Figure 1C). After capsule endoscope removal, the patient developed pneumonia in the peripheral airways, which improved after approximately 1 week of antibiotic treatment.

The timeline of the clinical course is shown in Table 1.

**TABLE 1:** Timeline of the clinical episode and therapeutic interventions.

Date/Period	Clinical events and interventions
Past medical history	Total gastrectomy, cholecystectomy and chronic obstructive pulmonary disease (COPD).
Day 1 (Event)	Swallowed a capsule endoscope. Immediately developed cough and pharyngeal discomfort. SpO2 was 94% on room air.
Day 1 (Diagnosis)	Chest x‐ray and CT confirmed the capsule endoscope impacted deep within the right basal segmental bronchus.
Day 1 (Intervention)	Initial assessment: Flexible bronchoscopy revealed complete impaction. Conventional forceps manipulation was impossible < br >. Step 1 (Fluoroscopy‐guided disimpaction): A guidewire was passed through a slight gap, and a balloon catheter was inserted and expanded with Gastrografin. The capsule was pulled towards the central airway < br >. Step 2 (Retrieval): The capsule was successfully extracted in the central airway using a retrieval loop net.
Postprocedure	Developed localised pneumonia in the peripheral airway.
Approx. 1 week later	Pneumonia improved following antibiotic treatment.

## 3 Discussion

This case is a rare instance in which a capsule endoscope impacted the peripheral airway due to aspiration and was safely and reliably removed using a two‐step strategy combined with fluoroscopy.

The small bowel capsule endoscope (26 × 11 mm) is larger than typical aspirated foreign bodies (e.g., acetaminophen 500 mg tablets (15 × 8 mm) and peanuts (20 × 10 mm)). Furthermore, it has a smooth surface to facilitate swallowing. This characteristic significantly hinders grasping with forceps. Since it is also nonmetallic, magnetic devices cannot be used and suction removal is physically unsuitable. That is, the capsule endoscope is a “special foreign body structurally unsuited for conventional foreign body removal techniques,” and the difficulty of removal becomes particularly pronounced when it is impacted in the peripheral airway.

Previous reports [3, 4, 5, 6, 7, 8, 9, 10, 11, 12, 13, 14, 15] frequently describe cases where aspirated capsule endoscopes remain mostly in the central airways, mainly the right main bronchus and reports indicate successful removal using bag‐like devices such as the Roth Net. A review by Thorndal et al. [1] also notes that 38% of the patients spontaneously coughed out, and a few patients migrated peripherally.

While previous reports [3, 4, 5, 6, 7, 8, 9, 10, 11, 12, 13, 14, 15] predominantly address central airway capsule aspirations, our comprehensive search yielded no cases detailing retrieval from a peripheral bronchus. The main innovation of our report is the effective management of a completely impacted peripheral scenario, where distal visualization is entirely obstructed, making standard techniques unsafe. By combining fluoroscopy with a balloon catheter, we established a novel rescue strategy that bridges this critical gap in airway management.

As a fundamental principle of bronchoscopic intervention, visualization distal to the obstruction/stenosis is the most crucial safety requirement to avoid perforation and airway injury. In this case, where the foreign body was completely impacted and visualization of the peripheral airway was impossible, a departure from standard indications was considered. In conditions where securing the visual field is difficult, supplementing positional information with x‐ray fluoroscopy becomes essential for safely manipulating devices. Fluoroscopy allows real‐time visualization of the device's position, direction of advancement and expansion status, making it a rational method to compensate for the limitations of bronchoscopy when the visual field is obscured.

Based on the background above, a two‐step strategy was employed in this case: (1) Disimpaction under fluoroscopy and (2) reliable retrieval in the central airway. In the first step, the positional relationship with the capsule trajectory was accurately assessed under fluoroscopy, and the balloon catheter was guided distally using the guidewire as a marker. The impaction was released by safely performing the traction manoeuvre under fluoroscopy. In the second step, the capsule, once guided to the central airway, was reliably captured with a bag‐like device, enabling retrieval without grasping its smooth surface. This strategy is significant because it provides a reproducible and safe approach for peripheral impaction, which is usually extremely difficult.

While this strategy is highly effective, it has several limitations. Technically, it fundamentally requires fluoroscopic equipment and a minimal anatomical gap between the capsule and the bronchial wall to allow initial guidewire passage. Furthermore, because the traction manoeuvre is performed without direct bronchoscopic visualization, there remains a theoretical risk of airway mucosal injury, necessitating careful manipulation. Finally, as a single case report, further accumulation of cases is needed to establish its universal safety. Nevertheless, given the extreme difficulty of retrieving peripherally impacted capsules, this fluoroscopy‐assisted two‐step approach remains a highly valuable rescue option when conventional bronchoscopic removal fails.

The number of capsule endoscopy procedures has increased in recent years, raising the possibility of encountering more aspiration cases in the future. The fluoroscopy‐assisted two‐step strategy presented in this case has high practical utility and broad applicability to peripheral impaction cases and could become valuable in future clinical practice.

In conclusion, we experienced a rare case of peripheral airway impaction due to capsule endoscope aspiration. For foreign bodies characterised by a smooth surface, large size and nonmetallic composition, a two‐step strategy—performing disimpaction under fluoroscopy guidance and then retrieving it in the central airway with a bag‐like device is effective. Especially in cases of peripheral impaction, the use of fluoroscopy as an adjunct to bronchoscopic visualization enables safe and reliable foreign body extraction.

Due to the patient's underlying delirium, it was not possible to directly obtain his personal perspective or verbal feedback regarding the treatments he received. However, from a clinical standpoint, the post‐aspiration coughing and pharyngeal discomfort resolved following the successful extraction of the capsule endoscope. Subsequently, the patient appeared calm and free from respiratory distress.

## Author Contributions

All listed authors contributed to the article.

## Funding

The authors have nothing to report.

## Consent

The authors declare that written informed consent was obtained for the publication of this manuscript and accompanying images. Consent was obtained from the patient in accordance with the form specified by the Independent Ethics Committee of Tohoku Medical and Pharmaceutical University Hospital. The authors attest that this consent form complies with the Journal requirements as outlined in the author guidelines.

## Conflicts of Interest

The authors declare no conflicts of interest.

## Data Availability

The data that support the findings of this study are available on request from the corresponding author. The data are not publicly available due to privacy or ethical restrictions.
